# On the Analysis of Regularized Fuzzy Systems of Uncertain Differential Equations

**DOI:** 10.3390/e25071010

**Published:** 2023-06-30

**Authors:** Anatoliy Martynyuk, Gani Stamov, Ivanka Stamova, Yulya Martynyuk-Chernienko

**Affiliations:** 1 S. P. Timoshenko Institute of Mechanics, NAS of Ukraine, 3 Nesterov Str., 03057 Kiev, Ukraine; center@inmech.kiev.ua (A.M.); centera@inmech.kiev.ua (Y.M.-C.); 2 Department of Mathematics, University of Texas at San Antonio, San Antonio, TX 78249, USA; gani.stamov@utsa.edu

**Keywords:** fuzzy differential equations, regularization correctness, successive approximation, continuity, global solutions, approximate solutions, autonomous fuzzy differential equations, stability analysis

## Abstract

This article analyzes a regularized set of fuzzy differential equations with respect to an uncertain parameter. We provide sufficient conditions for the correctness of a new regularization scheme. For the resulting family of regularized fuzzy differential equations, the following properties are analyzed, and efficient criteria are proposed: successive approximations, continuity, global existence of solutions, existence of approximate solutions, existence of solutions in the autonomous case. In addition, we develop stability criteria for the regularized family of fuzzy differential equations on the basis of the comparison technique and the method of nonlinear integral inequalities. We expect that the derived results will inspire future research work in this direction.

## 1. Introduction

If the equations of the perturbed motion of a mechanical or other natural system are derived on the basis of the classical Newton’s law, we obtain an idealized problem. Real-world processes and phenomena can be studied adequately from real content only if the uncertainty in their parameters and the fuzziness of the accepted model are considered as they are accepted by the researcher. The concept of fuzzy sets, introduced in 1965 by Zadeh [1], allowed the development of a general theory of fuzzy differential systems, which has been widely applied in many fields of the mathematical modeling of real-world processes. Several books and articles that summarized important results of the theory of fuzzy differential equations and their applications in electricity, mechanics, physics, engineering, biology, and economics have been indicated by the authors in [2] (see, for example, Refs. [3,4,5,6,7,8,9,10,11,12,13,14,15,16] and the references therein). Also, the notion of fuzzy entropy in the framework of fuzzy functions has recently engaged research interests [17,18]. In addition, different notions of entropies are applicable to fuzzy dynamical systems [19]. Any new contribution in the area of fuzzy systems will lead to future progress in their theory and expansions of their applications.

On the other hand, taking into account the inaccuracy of system parameters is an important step in their real applications. That is why numerous researchers study the behavior of systems with uncertain parameters [20,21,22,23,24,25,26,27].

However, hybrid models of fuzzy differential systems that involve uncertain parameters have been investigated very seldom [28,29,30]. The basic goal of our research is to develop this scientific area.

The main strategy applied in the investigation of systems with uncertain values of the parameters is that of robust analysis [21,22,23,31,32]. In this paper, we will use a different strategy that is based on a regularization process proposed in [2] for the fuzzy systems of differential equations with uncertain parameters that belong to a certain domain. Using the new technique, important fundamental properties of the solutions, such as the existence and estimated distance between solutions of the regularized differential equations, are discussed.

The key outcomes of our study are:

1. We apply a new regularization process in order to study a fuzzy system of differential equations with uncertain values of the parameter;
2. Efficient criteria for the correctness of the regularization process are derived;
3. Successive approximations for the regularized differential equation are considered;
4. New sufficient conditions for the continuous dependence of the solutions of the regularized equation of the initial data are established;
5. Conditions for the existence of an approximate solution are proposed;
6. Autonomous fuzzy differential equations are considered and criteria for the existence of their solutions are presented;
7. New stability criteria are proved based on the regularized fuzzy differential equations.

Here, the presentation is according to the following plan. Section 2 gives the basic concepts of the theory of fuzzy sets and functions that are required for further presentation. In Section 3 conditions for the correctness of the regularization process applied to the fuzzy differential equations are established. In Section 4, the construction of successive approximations for the regularized equation is discussed. Section 5 analyzes the continuity of the family of solutions of the regularized fuzzy differential equations and offers new sufficient conditions. Section 6 deals with global existence criteria. In Section 7 we investigate conditions for the existence of an approximate solution for the uncertain system of fuzzy differential equations. Section 8 considers the autonomous case. The existence of solutions for an autonomous fuzzy differential equation is analyzed. In Section 9, efficient conditions for the stability of a stationary state of the regularized differential equations are obtained. Finally, in Section 10 some comments and discussions are derived.

## 2. Nomenclature of Fuzziness

For the completeness of the presentation, we will state some details of fuzziness following [2,9,15].

In this paper, we denote by *X* a basic set. For any ξ∈X, a membership function ψ(ξ) takes its values from the closed interval [0,1].

For a fuzzy set with a membership function ψ on *X*, its ω-level sets ${\left[\psi \right]}^{\omega }$ are defined as

$${\left[\psi \right]}^{\omega }=\left\{\xi \in X:\;\psi \left(\xi \right)\geq \omega \right\}\;\mathrm{for}\;\mathrm{any}\;\omega \in \left(0,1\right]$$

and its support is defined by

$${\left[\psi \right]}^{0}=\bar{\underset{\omega \in (0,1]}{⋃}{\left[\psi \right]}_{\omega }}.$$

Consider two nonempty subsets Υ and Φ of $R^{n}$. Then, the Hausdorff distance between them is given as

$$d_{H}\left(\Upsilon ,\Phi \right)=\min\left\{r\geq 0:\Upsilon \subseteq \left\{\Phi \cup \Phi_{r}\left(0\right)\right\},\Phi \subseteq \left\{\Upsilon \cup \Phi_{r}\left(0\right)\right\}\right\},$$

where $\Phi_{r}\left(0\right)=\{\xi \in R^{n}:∥\xi ∥<r\},r\geq 0$.

Note that the above-defined Hausdorff distance $d_{H}\left(\Upsilon ,\Phi \right)$ is a metric for any nonempty closed sets in $R^{n}$.

The pair $\left(C^{n},d_{H}\right)$ is a metric space, where $C^{n}$ is the set of all nonempty closed sets in $R^{n}$.

In our research, we will also use the space $E^{n}$ of functions $\psi :R^{n}\to \left[0,1\right]$ that have the following properties [2]:

(1) ψ is upper semicontinuous in the sense of Baire [9,15,24];
(2) There exists a $\xi_{0}\in R^{n}$ such that $\psi (\xi_{0})=1$;
(3) ψ is fuzzy convex, i.e.,
  ψ(αξ+(1−α)η)≥min[ψ(ξ),ψ(η)]
  for any values of α∈[0,1];
(4) The closure of the set $\left\{\xi \in R^{n}:\psi \left(\xi \right)>0\right\}$ is a compact subset of $R^{n}$.

For two sets $\psi ,\phi \in E^{n}$, the metric in the space $E^{n}$ is defined [2] as follows

$$d\left(\psi ,\phi \right)=\sup\{|\psi \left(\xi \right)-\phi \left(\xi \right)|:\xi \in R^{n}\}.$$

The least upper bound of the metric *d* on the space $E^{n}$ is defined by

$$d\left[\psi ,\phi \right]=\sup\{d_{H}\left({\left[\psi \right]}^{\omega },{\left[\phi \right]}^{\omega }\right):\omega \in \left[0,1\right]\}$$

for $\psi ,\phi \in E^{n}$ and is a metric in $E^{n}$.

The symbol $P_{k}\left(R^{n}\right)$ denotes the family of all nonempty compact convex subsets of $R^{n}$.

Let T=[a,b],b>a>0 be a compact interval. Then, the integral of a mapping *U* on the interval *T* is denoted by $\int_{a}^{b}U\left(t\right)dt$ and is defined as

$$\int_{T}U\left(t\right)dt=\left\{\int_{T}\bar{u}\left(t\right)dt|\bar{u}:\;T\to R^{n}\mathrm{is}a\mathrm{measurable}\mathrm{selection}\mathrm{for}U_{\omega }\right\}$$

for any 0<ω≤1.

Next, let $\psi ,\phi \in E^{n}$ where there exists $\zeta \in E^{n}$ such that ϕ=ϕ+ζ. Then, ζ is called the Hukuhara difference of the subsets ψ and ϕ and is denoted by ψ−ϕ.

We will say that the mapping $U:\;T\to E^{n}$ is differentiable at the point $t_{0}\in T$. If the value $U^{\prime }\left(t_{0}\right)$ exists, $U^{\prime }\left(t_{0}\right)\in E^{n}$ is such that both limits

$$\lim\left\{\left[U\left(t_{0}+\chi \right)-U\left(t_{0}\right)\right]\chi^{-1}:\chi \to 0^{+}\right\}\mathrm{and}\lim\left\{\left[U\left(t_{0}\right)-U\left(t_{0}-\chi \right)\right]\chi^{-1}:\chi \to 0^{+}\right\}$$

exist and are equal to $U^{\prime }\left(t_{0}\right)$. The above limits are considered in the metric space $\left(E^{n},d\right)$.

Note that the family $\left\{D_{H}U_{\omega }\left(t\right):\omega \in \left[0,1\right]\right\}$ determines an element $U^{\prime }\left(t\right)\in E^{n}$. If $F:T\to E^{n}$ is differentiable at t∈T, then the element $U^{\prime }(t)$ is called the fuzzy derivative of U(t) at the point *t*.

If $U_{\omega }$ is differentiable, the mapping $U_{\omega }$ is differentiable in the sense of Hukuhara for all ω∈[0,1] and

$$D_{H}U_{\omega }\left(t\right)={\left[U^{\prime }\left(t\right)\right]}^{\omega },$$

where $D_{H}U_{\omega }$ is the Hukuhara-type derivative of $U_{\omega }$.

More detailed information from the theory of fuzzy sets and functions is available in Refs. [9,15,24,33,34] and some others.

## 3. Conditions for Correct Regularization

In our paper [2], we proposed a regularization scheme for a system of fuzzy differential equations with respect to an uncertain parameter. In this section, we will offer criteria for the correctness of the regularization.

Consider the following fuzzy system of differential equations with an uncertain parameter

(1) $$\frac{d\psi }{dt}=F\left(t,\psi ,\lambda \right),\;\psi \left(t_{0}\right)=\psi_{0},$$

where $\psi \in E^{n}$, $F\in C(R_{+}\times E^{n}\times P,E^{n})$, λ∈P is an uncertain parameter, and P is a compact set in $R^{k}$.

Next, along with System (1), we analyze the initial value problem (IVP) for a system of fuzzy differential equations of the type

(2) $$\frac{d\phi }{dt}=F_{\omega }\left(t,\phi \right),\;\phi \left(t_{0}\right)=\phi_{0},$$

where $F_{\omega }\in C\left(I\times E^{n},E^{n}\right)$, $I=[t_{0},t_{0}+a]$, $t_{0}\geq 0$, a>0, and ω∈[0,1].

The family of mappings $F_{\omega }\left(t,\phi \right)$ in (2) is defined by

(3) $$F_{\omega }\left(t,\phi \right)=F_{M}\left(t,\phi \right)\omega +\left(1-\omega \right)F_{m}\left(t,\phi \right),\;0\leq \omega \leq 1,$$

where

(4) $$F_{m}\left(t,\phi \right)=\bar{\mathrm{co}}\underset{\lambda \in P}{⋂}F\left(t,\phi ,\lambda \right),\;P\subseteq R^{k},$$

(5) $$F_{M}\left(t,\phi \right)=\bar{\mathrm{co}}\underset{\lambda \in P}{⋃}F\left(t,\phi ,\lambda \right),\;P\subseteq R^{k}.$$

Here, and in what follows, we assume that $F_{m}\left(t,\phi \right)$, $F_{M}\left(t,\phi \right)\in R_{+}\times E^{n}$.

The family of fuzzy differential Equation (2) is said to be regularized on the uncertain parameter λ∈P with respect to System (1).

The solutions of the IVP (2) are the family of mappings $\phi :I\to E^{n}$ [2], which are weakly continuous and such that

$$\phi \left(t\right)=\phi_{0}+\int_{t_{0}}^{t}F_{\omega }\left(s,\phi \left(s\right)\right)ds$$

for all t∈I and any value of ω∈[0,1].

**Definition** **1.** *The regularization schemes (3)–(5) of the fuzzy differential equation with uncertain parameter (1) aer said to be correct. If for any β>0, there exists 0<γ(β)<β such that*

(6) $$d\left[\psi_{0},\phi_{0}\right]<\gamma \left(\beta \right)$$

*implies*

(7) d[ψ(t),ϕ(t)]<β

*for all ω∈[0,1] and $t\geq t_{0}$, for which the corresponding solutions ψ(t) and ϕ(t) of the fuzzy differential models (1) and (2) exist.*

If the inequality (7) is satisfied for at least one pair of corresponding values of λ∈P and $\omega^{*}\in \left[0,1\right]$, then the regularization is said to be weak.

In the following, we will propose criteria for the correct regularization of the fuzzy differential system (1).

**Lemma** **1.** *Assume that:*

*(1) The corresponding solution ψ(t) of (1) exists on $t\geq t_{0}$ for any λ∈P.*

*(2) The corresponding solution ϕ(t) of (2) exists on $t\geq t_{0}$ for any ω∈[0,1].*

*(3) A function $L\left(t\right)\in C\left(R_{+},R_{+}\right)$ exists such that*

$$d[F\left(t,\psi ,\lambda \right),F_{\omega }\left(t,\phi \right)]\leq L\left(t\right)d\left[\psi ,\phi \right]$$

*for all values of λ∈P, ω∈[0,1] and any $\psi ,\phi \in E^{n}$.*

*(4) For $t\in [t_{0},\infty )$, the following inequality*

$$\int_{t_{0}}^{t}L\left(s\right)ds\leq \ln\frac{\beta }{\gamma }$$

*is satisfied.*

*Then, the regularization process (3)–(5) is correct in the sense of Definition 1.*

**Proof.**  The relations

$$\psi \left(t\right)=\psi_{0}+\int_{t_{0}}^{t}F\left(s,\psi \left(s\right),\lambda \right)ds$$

and

$$\phi \left(t\right)=\phi_{0}+\int_{t_{0}}^{t}F_{\omega }\left(s,\phi \left(s\right)\right)ds$$

imply the estimate

(8) $${℘}_{\omega }\left(t\right)\leq {℘}_{\omega }\left(t_{0}\right)+\int_{t_{0}}^{t}L\left(s\right){℘}_{\omega }\left(s\right)ds,$$

where ${℘}_{\omega }\left(t\right)=d\left[\psi \left(t\right),\phi \left(t\right)\right]$.

Now, the statement of Lemma 1 follows from (8) and Corollary 5.4 in [2]. □

**Corollary** **1.** *If Condition (3) in Lemma 1 is satisfied for a constant $L_{0}>0$ on a finite interval $\left[t_{0},t_{0}+a\right]$, then the assertion of Lemma 1 holds whenever*

$$0<L_{0}\leq \frac{1}{a}\ln\frac{\beta }{\gamma }$$

*for any β>0 and 0<γ(β)<β.*

## 4. Successive Approximations

In this section, we consider the IVP (2) under more general conditions than the Lipschitz-type condition, and we provide successive approximation criteria.

Denote by $S\left(\phi_{0},b\right)=\left\{\phi \in E^{n}:d\left[\phi ,\phi_{0}\right]\leq b\right\}$, and consider the family of functions $F_{\omega }\left(t,\phi \right)$ on the domain $\Phi =I\times S(\phi_{0},b)$ for all ω∈[0,1].

**Theorem** **1.** *Assume that:*

*(1) For any ω∈[0,1], the family $F_{\omega }\left(t,\phi \right)\in C\left(\Phi ,E^{n}\right)$, and there exist constants $K_{0}\left(\omega \right)$ such that $d\left[F_{\omega }\left(t,\phi \right),\theta_{0}\right]\leq K_{0}\left(\omega \right)$ on Φ, where the state $\theta_{0}\in E^{n}$ is defined as*

$$\theta_{0}\left(x\right)=\left\{\begin{array}{cc} 1, & \mathrm{for}\xi =0,\\ 0, & \mathrm{for}\xi \in R^{n}\setminus \left\{0\right\}. \end{array}\right.$$

*(2) There exists a continuous on I×[0,2b] real function G(t,η) and a constant $K_{1}$ such that $0\leq G\left(t,\eta \right)\leq K_{1}$ for (t,η)∈I×[0,2b], G(t,0)=0 for t∈I, G(t,η) is nondecreasing with respect to η for any t∈I, and η(t)=0 is the unique solution of the IVP*

(9) $$\frac{d\eta }{dt}=G\left(t,\eta \right),\;\eta \left(t_{0}\right)=0.$$

*(3) For any ω∈[0,1], the following estimate*

$$d\left[F_{\omega }\left(t,\phi \right),F_{\omega }\left(t,\bar{\phi }\right)\right]\leq G\left(t,d\left[\phi ,\bar{\phi }\right]\right)$$

*holds for $\left(t,\phi \right),\left(t,\bar{\phi }\right)\in \Phi$.*

*Then, there exist successive approximations in the form*

(10) $$\phi_{n+1}\left(t\right)=\phi_{0}+\int_{t_{0}}^{t}F_{\omega }\left(s,\phi_{n}\left(s\right)\right)ds,\;n=0,1,2,\ldots ,$$

*for $t\in [t_{0},t_{0}+\Delta ]$, where Δ=min{a,b/K}, $K=\max\{{\bar{K}}_{0},K_{1}\}$, ${\bar{K}}_{0}=\max_{\omega }K_{0}\left(\omega \right)$, and the sequence of uniformly continuous functions is uniformly convergent to the unique solution ϕ(t) of the IVP (2) on $\left[t_{0},t_{0}+\Delta \right]$.*

**Proof.**  From Condition (1) of Theorem 1 and (10), we have

$$d\left[\phi_{n+1}\left(t\right),\phi_{0}\right]=d\left[\phi_{0}+\int_{t_{0}}^{t}F_{\omega }\left(s,\phi_{n}\left(s\right)\right)ds,\phi_{0}\right]$$

$$=d\left[\int_{t_{0}}^{t}F_{\omega }\left(s,\phi_{n}\left(s\right)\right)ds,\theta_{0}\right]\leq \int_{t_{0}}^{t}d\left[F_{\omega }\left(s,\phi_{n}\left(s\right)\right),\theta_{0}\right]ds$$

(11) $$\leq K_{0}\left(\omega \right)\left(t-t_{0}\right)=K_{0}\left(\omega \right)a\leq b,$$

where $a=t-t_{0}$ for $t_{0}\geq 0$, and hence, the functions $\left\{\phi_{n}\left(t\right)\right\}$ are defined on $\left[t_{0},t_{0}+\Delta \right]$ for any ω∈[0,1].

For (9), we define the successive approximations as follows:

$$\eta_{0}\left(t\right)=K\left(t-t_{0}\right),$$

$$\eta_{n+1}\left(t\right)=\int_{t_{0}}^{t}G\left(s,\eta_{n}\left(s\right)\right)ds,t_{0}\leq t\leq t_{0}+\Delta ,\;n=0,1,2,\ldots .$$

We can observe that

$$0\leq \eta_{n+1}\left(t\right)\leq \eta_{n}\left(t\right)\mathrm{for}t\in \left[t_{0},t_{0}+\Delta \right].$$

Since $|\eta_{n}^{\prime }\left(t\right)|\leq G\left(t,\eta_{n-1}\left(t\right)\right)\leq K_{1}$, by the Arzela–Ascoli Theorem, we have $\lim_{n\to \infty }\eta_{n}\left(t\right)=\eta \left(t\right)$ uniformly on $t\in [t_{0},t_{0}+\Delta ]$, i.e., the sequence $\left\{\eta_{n}\left(t\right)\right\}$ is monotonic. The function η(t) satisfies the equation in the IVP (9), and according to Condition (2) of Theorem 1, it is such that η(t)≥0 for all $t\in [t_{0},t_{0}+\Delta ]$.

From (11), it follows that

$$d\left[\phi_{1}\left(t\right),\phi_{0}\right]\leq \int_{t_{0}}^{t}d\left[F_{\omega }\left(s,\phi_{0}\right),\theta_{0}\right]ds\leq K\left(t-t_{0}\right)=\eta_{0}\left(t\right)$$

for all ω∈[0,1].

Let it be that for some *k*, we have

$$d\left[\phi_{k}\left(t\right),\phi_{k-1}\left(t\right)\right]\leq \eta_{k-1}\left(t\right)\;\mathrm{on}\;\left[t_{0},t_{0}+\Delta \right].$$

From the inequality

$$d\left[\phi_{k+1}\left(t\right),\phi_{k}\left(t\right)\right]\leq \int_{t_{0}}^{t}d\left[F_{\omega }\left(s,\phi_{k}\left(s\right)\right),F_{\omega }\left(s,\phi_{k-1}\left(s\right)\right)\right]ds,$$

Condition (3) of Theorem 1 and the fact that G(t,η) is nondecreasing with respect to η, we obtain

(12) $$\begin{array}{ccc} d[\phi_{k+1}\left(t\right),\phi_{k}\left(t\right)] & \leq & \int_{t_{0}}^{t}G\left(s,d\left[\phi_{k}\left(s\right),\phi_{k-1}\left(s\right)\right]\right)ds\\ & \leq & \int_{t_{0}}^{t}G\left(s,\eta_{k-1}\left(s\right)\right)ds=\eta_{k}\left(t\right). \end{array}$$

From (12), by induction, we obtain

$$d\left[\phi_{n+1}\left(t\right),\phi_{n}\left(t\right)\right]\leq \eta_{n}\left(t\right),t\in \left[t_{0},t_{0}+\Delta \right]$$

for any n=0,1,2,…, and ω∈[0,1].

Set $\Omega \left(t\right)=d[\phi_{n+1}\left(t\right),\phi_{n}\left(t\right)]$ for $t\in [t_{0},t_{0}+\Delta ]$. Then, for the upper derivative $D^{+}\Omega \left(t\right)$, we have

(13) $$D^{+}\Omega \left(t\right)\leq G\left(t,d\left[\phi_{n}\left(t\right),\phi_{n-1}\left(t\right)\right]\right)\leq G\left(t,\eta_{n-1}\left(t\right)\right).$$

Let $n_{1}\leq n_{2}$, $n_{1},n_{2}=0,1,2,\ldots$. For the distance between $\phi_{n_{1}}^{\prime }\equiv d\phi_{n_{1}}/dt$ and $\phi_{n_{2}}^{\prime }\equiv d\phi_{n_{2}}/dt$, we have

$$d[\phi_{n_{1}}^{\prime }\left(t\right),\phi_{n_{2}}^{\prime }\left(t\right)]$$

$$=d\left[F_{\omega }\left(t,\phi_{n_{1}-1}\left(t\right)\right),F_{\omega }\left(t,\phi_{n_{2}-1}\left(t\right)\right)\right]\leq d\left[F_{\omega }\left(t,\phi_{n_{1}}\left(t\right)\right),F_{\omega }\left(t,\phi_{n_{1}-1}\left(t\right)\right)\right]$$

$$+d\left[F_{\omega }\left(t,\phi_{n_{1}-1}\left(t\right)\right),F_{\omega }\left(t,\phi_{n_{2}-1}\left(t\right)\right)\right]+d\left[F_{\omega }\left(t,\phi_{n_{2}}\left(t\right)\right),F_{\omega }\left(t,\phi_{n_{2}-1}\left(t\right)\right)\right]$$

(14) $$\leq G\left(t,\eta_{n_{1}-1}\left(t\right)\right)+G\left(t,\eta_{n_{2}-1}\left(t\right)\right)+G\left(t,d\left[\phi_{n_{1}}\left(t\right),\phi_{n_{2}}\left(t\right)\right]\right)$$

for any ω∈[0,1]. Then, (14) implies that

$$D^{+}\Omega \left(t\right)\leq d\left[\phi_{n_{1}}^{\prime }\left(t\right),\phi_{n_{2}}^{\prime }\left(t\right)\right]\leq G\left(t,\Omega \left(t\right)\right)+2G\left(t,\eta_{n_{1}-1}\left(t\right)\right),\;t\in \left[t_{0},t_{0}+\Delta \right].$$

Since G(t,η) is a nondecreasing with respect to the η function, then $\eta_{n_{2}-1}\leq \eta_{n_{1}-1}$ for any $n_{1}\leq n_{2}$, i.e., the sequence $\left\{\eta_{n}\left(t\right)\right\}$, is nonincreasing. Theorem 1.4.1 from [33] guarantees that

$$\Omega \left(t\right)\leq \sigma_{n}\left(t\right),\;t\in \left[t_{0},t_{0}+\Delta \right],$$

for n=0,1,2,…, where $\sigma_{n}\left(t\right)$ is the maximal solution of the IVP

(15) $$d\sigma_{n}/dt=G\left(t,\sigma_{n}\right)+2G\left(t,\eta_{n-1}\left(t\right)\right),\sigma_{n}\left(t_{0}\right)=0.$$

Since $2G(t,\eta_{n-1}\left(t\right))\to 0$ as n→∞ uniformly on $\left[t_{0},t_{0}+\Delta \right]$, then $\sigma_{n}\left(t\right)\to 0$ uniformly on $\left[t_{0},t_{0}+\Delta \right]$. Hence, the sequence of functions $\left\{\phi_{n}\left(t\right)\right\}$ converges to the solution ϕ(t) of the IVP (2) uniformly on $t\in [t_{0},t_{0}+\Delta ]$ for any ω∈[0,1].

In order to prove the uniqueness of the limit, we will study another solution $\phi^{0}\left(t\right)$ of the IVP (2). Set $g\left(t\right)=d[\phi \left(t\right),\phi^{0}\left(t\right)]$ and $g(t_{0})=0$. From $D^{+}g\left(t\right)\leq G\left(t,g\left(t\right)\right)$ for any t∈I as $g\left(t\right)\leq \sigma \left(t,t_{0},0\right)$ for t∈I, it follows that $\phi \left(t\right)=\phi^{0}\left(t\right)$ for all t∈I and ω∈[0,1].

The proof is completed. □

## 5. Continuity Concept

In this section, we first establish the following result.

**Lemma** **2.** *Assume that:*

*(1) For any ω∈[0,1], the family $F_{\omega }\in C\left(I\times E^{n},E^{n}\right)$ and*

$$G_{\omega }\left(t,\sigma \right)=\max_{d[\phi ,\phi_{0}]\leq \sigma }d\left[F_{\omega }\left(t,\phi \right),\theta_{0}\right].$$

*(2) For any ω∈[0,1], the maximal solution $\sigma_{\omega }\left(t,t_{0},0\right)$ of the family of equations*

(16) $$d\eta /dt=G_{\omega }\left(t,\eta \right),\eta \left(t_{0}\right)=\eta_{0}=0$$

*exists on I.*

*Then*

$$d\left[\phi \left(t,t_{0},0\right),\theta_{0}\right]\leq \sigma_{\omega }\left(t,t_{0},0\right)$$

*for t∈I and any ω∈[0,1].*

**Proof.**  Let denote $g\left(t\right)=d[\phi \left(t,t_{0},0\right),\phi_{0}]$ for t∈I and ω∈[0,1]. From the conditions of Lemma 2, we have

$$D^{+}g\left(t\right)\leq d\left[d\eta \left(t,t_{0},0\right)/dt,\theta_{0}\right]=d\left[F_{\omega }\left(t,\phi \left(t,t_{0},0\right)\right),\theta_{0}\right]$$

$$\leq \max_{d[\phi ,\phi_{0}]\leq \sigma }d\left[F_{\omega }\left(t,\phi \right),\theta_{0}\right]=G_{\omega }\left(t,g\left(t\right)\right).$$

Now, for the IVP (16), we will apply Theorem 4.1 from [33] to obtain

$$g\left(t\right)\leq d\left[\phi \left(t,t_{0},0\right),\theta_{0}\right]\leq \sigma_{\omega }\left(t,t_{0},0\right)$$

for any ω∈[0,1] and t∈I. □

**Theorem** **2.** *If, in addition to the conditions of Theorem 1, the family of solutions $\sigma_{\omega }\left(t,t_{0},0\right)$ of (16) is continuous with respect to $\left(t_{0},\eta_{0}\right)$ for any ω∈[0,1], then the family of solutions $\phi (t,t_{0},\phi_{0})$ of (2) is continuous with respect to $\left(t_{0},\phi_{0}\right)$ for any value of ω∈[0,1].*

**Proof.**  Let $\phi (t,t_{0},\phi_{0})$ and $\bar{\phi }\left(t,t_{0},{\bar{\phi }}_{0}\right)$ be any two solutions of (2) with initial data, respectively, $\left(t_{0},\phi_{0}\right)$ and $\left(t_{0},{\bar{\phi }}_{0}\right)$. We have from Theorem 1 that

$$d\left[\phi \left(t,t_{0},\phi_{0}\right),\bar{\phi }\left(t,t_{0},{\bar{\phi }}_{0}\right)\right]\leq \sigma^{*}\left(t,t_{0},d\left[\phi_{0},{\bar{\phi }}_{0}\right]\right)$$

for any t∈I and ω∈[0,1], where $\sigma^{*}\left(t,t_{0},0\right)=\max_{\omega }\sigma_{\omega }\left(t,t_{0},0\right)$. Since uniformly on t∈I, $\lim_{\psi_{0}\to \phi_{0}}r^{*}\left(t,t_{0},d\left[\phi_{0},{\bar{\phi }}_{0}\right]\right)$$=\sigma^{*}\left(t,t_{0},0\right)$ and $\sigma^{*}\left(t,t_{0},0\right)\equiv 0$, we have $\lim_{\phi_{0}\to {\bar{\phi }}_{0}}d[\phi \left(t,t_{0},\phi_{0}\right)$, $\bar{\phi }\left(t,t_{0},{\bar{\phi }}_{0}\right)]=0$. Hence, $\phi (t,t_{0},\phi_{0})$ is continuous with respect to $\phi_{0}$ for any ω∈[0,1]. The continuity with respect to $t_{0}$ of the family of solutions $\phi (t,t_{0},\phi_{0})$ follows from Lemma 2 considering the families of solutions $\phi (t,t_{0},\phi_{0})$ and $\bar{\phi }\left(t,\tau_{0},{\bar{\phi }}_{0}\right)$, where $\tau_{0}\geq t_{0}$, ω∈[0,1]. □

## 6. Global Existence

This section will be devoted to the global existence criteria for the solutions of the family of fuzzy differential Equation (2) for $t\geq t_{0}$.

**Theorem** **3.** *For the family of fuzzy differential Equation (2) assume that:*

*(1) For any ω∈[0,1], the set of functions $F_{\omega }\in C\left(R_{+}\times E^{n},E^{n}\right)$.*

(2) A family of functions $G_{\omega }^{*}\in C\left(R_{+}\times R_{+},R\right)$ exists such that $G_{\omega }^{*}\left(t,\eta \right)$ are nondecreasing with respect to η for $t\in R_{+}$, and

$$d\left[F_{\omega }\left(t,\phi \right),\theta_{0}\right]\leq G_{\omega }^{*}\left(t,d\left[\phi ,\theta_{0}\right]\right)$$

*for $\left(t,\phi \right)\in R_{+}\times E^{n}$ and ω∈[0,1].*

*(3) The maximal solution $\sigma_{\varkappa }\left(t,t_{0},\eta_{0}\right)$ of the IVP*

(17) $$d\eta /dt=G_{\omega }^{*}\left(t,\eta \right),\eta \left(t_{0}\right)=\eta_{0}\geq 0,$$

*exists for $t\geq t_{0}$ and any ω∈[0,1].*

*Then, if the local solution $\phi (t,t_{0},\phi_{0})$ of the IVP (2) exists, its maximal interval of existence for initial value $\phi_{0}$ such that $d\left[\phi_{0},\theta_{0}\right]\leq \eta_{0}$ is $\left[t_{0},\infty \right)$ for any ω∈[0,1].*

**Proof.**  Let the set of solutions $\phi (t,t_{0},\phi_{0})$ of the IVP (2) for values of ω∈[0,1] with initial data $\phi_{0}$ such that $d\left[\phi_{0},\theta_{0}\right]\leq \eta_{0}$ exists on the interval $\left[t_{0},b\right)$, 0<b<∞, and is not continuable to the right of *b*. For $g\left(t\right)=d[\phi \left(t,t_{0},\phi_{0}\right),\theta_{0}]$, from Lemma 2, we obtain

(18) $$g\left(t\right)\leq \bar{\sigma }\left(t,t_{0},\eta_{0}\right)$$

for all $t_{0}\leq t<b$, where $\bar{\sigma }\left(t,t_{0},\eta_{0}\right)=\max_{\omega }\sigma_{\omega }\left(t,t_{0},\eta_{0}\right)$. Next, for any two $t_{1},t_{2}\in \left[t_{0},b\right)$, $t_{1}<t_{2}$, the following estimate

$$d\left[\phi \left(t_{1}\right),\phi \left(t_{2}\right)\right]=d\left[\phi_{0}+\int_{t_{0}}^{t_{1}}F_{\omega }\left(s,\phi \left(s\right)\right)ds,\phi_{0}+\int_{t_{0}}^{t_{2}}F_{\omega }\left(s,\phi \left(s\right)\right)ds\right]$$

$$=d\left[\int_{t_{1}}^{t_{2}}F_{\omega }\left(s,\phi \left(s\right)\right)ds,\theta_{0}\right]\leq \int_{t_{1}}^{t_{2}}d\left[F_{\omega }\left(s,\phi \left(s\right)\right),\theta_{0}\right]ds$$

(19) $$\leq \int_{t_{1}}^{t_{2}}G_{\omega }^{*}\left(s,d\left[\phi ,\theta_{0}\right]\right)ds$$

holds for ω∈[0,1]. From Condition (3) of Theorem 3 and (19), we obtain

(20) $$d\left[\phi \left(t_{1}\right),\phi \left(t_{2}\right)\right]\leq \int_{t_{1}}^{t_{2}}G_{\omega }^{*}\left(s,\bar{\sigma }\left(s,t_{0},\eta_{0}\right)\right)ds=\bar{\sigma }\left(t_{2},t_{0},\eta_{0}\right)-\bar{\sigma }\left(t_{1},t_{0},\eta_{0}\right)$$

for any ω∈[0,1].

According to the assumptions of Theorem 3, the limit $\lim_{t\to b^{-}}\bar{\sigma }\left(t,t_{0},\eta_{0}\right)$ exists and is finite. Hence, for $t_{1},t_{2}\to b^{-}$ the limit $\lim_{t\to b^{-}}\phi \left(t,t_{0},\phi_{0}\right)$ exists for any ω∈[0,1]. In this case, we have $\lim_{t\to b^{-}}\phi \left(t,t_{0},\phi_{0}\right)=\phi \left(b,t_{0},\phi_{0}\right)=\phi \left(b\right)$.

We transform the IVP (2) in the form

(21) $$d\phi /dt=F_{\omega }\left(t,\phi \right),\phi \left(b\right)=\phi \left(b,t_{0},\phi_{0}\right).$$

Since the local solution $\phi (t,t_{0},\phi_{0})$ exists, then the solution of the IVP (21) can be continued to the right of *b*, which contradicts our assumption. The contradiction obtained shows that the family of solutions $\phi (t,t_{0},\phi_{0})$ exists for any $t\geq t_{0}$ whenever $\phi_{0}:d\left[\phi_{0},\theta_{0}\right]\leq \eta_{0}$ and conditions of Theorem 3 are met. □

**Remark** **1.** If there is at least one value $\omega^{*}\in \left[0,1\right]$ for which the conditions of Theorem 3 are true, then its assertion remains valid.

## 7. Approximate Solutions

It is clear that the fuzzy differential Equation (1) is approximated by the family of regularized differential Equation (2). Therefore, the problem of construction of approximate solutions of the class of fuzzy differential Equation (1) based on the family of solutions of the set of differential Equation (2) is of great interest.

We will introduce the following definition.

**Definition** **2.** A family of functions $\psi_{\varkappa }\left(t\right)\in C\left(R_{+},E^{n}\right)$ is called an ε-approximate solution of the family of fuzzy differential Equation (1). If for any ε>0, there is $\omega^{*}\in \left[0,1\right]$ with $d[\psi \left(t\right),\psi_{\omega^{*}}\left(t\right)]\leq \varepsilon$ for $t\geq t_{0}$.

**Remark** **2.** *Definition 2 is different from the definition of approximate solutions given in [9]. It is appropriate for the new theory based on the regularization process which is applied in our analysis.*

**Theorem** **4.** *Assume that:*

*(1) For any value of ω∈[0,1], the mappings $F_{\omega }\in C\left(R_{+}\times E^{n},E^{n}\right)$, and for any parameter, λ∈S the mapping $F\in C(R_{+}\times E^{n}\times P,E^{n})$.*

*(2) The family of functions $G_{\varkappa }\left(t,\eta \right)\in C\left(R_{+}\times R_{+},R\right)$ are nondecreasing with respect to η and such that*

$$d\left[F\left(t,\psi ,\lambda \right),F_{\omega }\left(t,\phi \right)\right]\leq G_{\omega }\left(t,d\left[\psi ,\phi \right]\right)$$

*for any ω∈[0,1] and all λ∈P.*

*(3) The family of the IVPs*

(22) $$d\eta /dt=G_{\omega }\left(t,\eta \right),\eta \left(t_{0}\right)=\eta_{0}\geq 0,$$

*has a maximal solution $\sigma_{\omega }\left(t,t_{0},\eta_{0}\right)$ defined for $t\geq t_{0}$.*

(4) There exists at least one value $\omega^{*}\in \left[0,1\right]$ such that $0<\sigma_{\omega^{*}}\left(t,t_{0},\eta_{0}\right)<\varepsilon$ for $t\geq t_{0}$.

Then, $\psi_{\omega^{*}}\left(t\right)$ is an ε-approximate solution of the IVP (1), whenever $d\left[\psi_{0},\phi_{0}\right]\leq \eta_{0}$.

**Proof.**  Let g(t)=d[ψ(t),ϕ(t)], where $\phi \left(t\right)=\psi_{\omega }\left(t\right)$ for any ω∈[0,1]. From the integral representations

$$\psi \left(t\right)=\psi_{0}+\int_{t_{0}}^{t}F\left(s,\psi \left(s\right),\lambda \right)ds$$

and

$$\phi \left(t\right)=\phi_{0}+\int_{t_{0}}^{t}F_{\omega }\left(s,\phi \left(s\right)\right)ds,$$

we have that for any ω∈[0,1]

$$g\left(t\right)=d\left[\psi_{0}+\int_{t_{0}}^{t}F\left(s,\psi \left(s\right),\lambda \right)ds,\phi_{0}+\int_{t_{0}}^{t}F_{\omega }\left(s,\phi \left(s\right)\right)ds\right]$$

$$\leq d\left[\psi_{0},\phi_{0}\right]+d\left[\int_{t_{0}}^{t}F\left(s,\psi \left(s\right),\lambda \right)ds,\int_{t_{0}}^{t}F_{\omega }\left(s,\phi \left(s\right)\right)ds\right]$$

$$\leq d\left[\psi_{0},\phi_{0}\right]+\int_{t_{0}}^{t}d\left[F\left(s,\psi \left(s\right),\lambda \right),F_{\omega }\left(s,\phi \left(s\right)\right)\right]ds\leq d\left[\psi_{0},\phi_{0}\right]$$

(23) $$+\int_{t_{0}}^{t}G_{\omega }\left(s,d\left[\psi \left(s\right),\phi \left(s\right)\right]\right)ds\leq \eta_{0}+\int_{t_{0}}^{t}G_{\omega }\left(s,g\left(s\right)\right)ds.$$

We now apply Theorem 1.6.1 from [35] to (23), and obtain

(24) $$g\left(t\right)\leq \sigma_{\omega }\left(t,t_{0},\eta_{0}\right)\mathrm{for}t\geq t_{0}.$$

It follows from (24) and Condition (4) of Theorem 4 that $\psi_{\omega^{*}}\left(t\right)$ for $\omega^{*}\in \left[0,1\right]$ is an ε-approximate solution of the IVP (1). □

## 8. Autonomous Uncertain Fuzzy Differential Equations

This section is devoted to the autonomous case. We consider an IVP for a class of autonomous fuzzy differential equations of the type

(25) $$\begin{array}{c} d\psi /dt=f\left(\psi ,\lambda \right),\;\psi \left(0\right)=\psi_{0}, \end{array}$$

where $f\in C(E^{n}\times P,E^{n})$ and λ∈P is the uncertain parameter. Define the mappings:

$$f_{m}\left(\psi \right)=\bar{\mathrm{co}}\underset{\lambda \in P}{⋂}f\left(\psi ,\lambda \right),$$

$$f_{M}\left(\psi \right)=\bar{\mathrm{co}}\underset{\lambda \in P}{⋃}f\left(\psi ,\lambda \right),$$

$$f_{\omega }\left(\psi \right)=\omega f_{m}\left(\psi \right)+\left(1-\omega \right)f_{M}\left(\psi \right),\;\omega \in \left[0,1\right].$$

Then, for ω∈[0,1], the regularized IVP for the IVP (25) has the form

(26) $$\begin{array}{c} d\phi /dt=f_{\omega }\left(\phi \right),\;\phi \left(0\right)=\phi_{0}, \end{array}$$

where $f_{\omega }\in C\left(E^{n},E^{n}\right)$ for all ω∈[0,1].

**Remark** **3.** *The fuzzy differential Equation (25) and the family of fuzzy differential Equation (26) are the autonomous versions of (1) and (2), respectively. In this autonomous case, the uncertain parameter λ∈P is used in the same sense for the regularization process of the fuzzy differential Equation (25) as in the regularization of (1).*

The following results are based on the estimation technique introduced in [9].

**Theorem** **5.** Assume that for $\omega^{*}\in \left[0,1\right]$:

*(1) There exists a constant β>0 such that*

$$\lim\sup\{\left[d\left[\phi +\chi f_{\omega^{*}}\left(\phi \right),\gamma +\chi f_{\omega^{*}}\left(\gamma \right)\right]-d\left[\phi ,\gamma \right]\right]\chi^{-1}:\chi \to 0^{+}\}\leq -\beta d\left[\phi ,\gamma \right]$$

*for $\phi ,\gamma \in E^{n}$ and*

(2) There exist constants L>0 and $K_{\omega^{*}}>0$ such that

$$d\left[f_{\omega^{*}}\left(w\right),\theta_{0}\right]\leq K_{\omega^{*}},$$

whenever $d[\phi ,\theta_{0}]\leq L$ and $\omega =\omega^{*}$.

*(3) For any $\phi_{0}\in E^{n}$, there exists a local solution of the IVP (26) on [0,a].*

Then, for any $\phi_{0}$ and $\omega =\omega^{*}\in \left[0,1\right]$, there exists a unique solution $w_{\omega }\left(t,\phi_{0}\right)$ of the IVP (26) on [0,∞).

**Proof.**  According to condition (3) of Theorem 5, there exists a value $\omega^{*}\in \left[0,1\right]$, for which the IVP (26) has a local solution on [0,a]. Denote the local solution by $\phi_{\omega }\left(t\right)=\phi_{\omega }\left(t,\phi_{0}\right)$ for some $\phi_{0}\in E^{n}$. Suppose that it exists *c*, 0<c<∞, such that the solution $\phi_{\omega }\left(t,\phi_{0}\right)$ exists on [0,c) and is not continuable to the right of *c*. Let for χ>0 the Hukuhara difference $\phi_{\omega }\left(t+\chi \right)-\phi_{\omega }\left(t\right)$ exists. Then, for $g\left(t\right)=d[\phi_{\omega }\left(t\right),\phi_{0}]$ and for t∈[0,c) we have

$$g\left(t+h\right)=d\left[\phi_{\omega }\left(t+\chi \right)-\phi_{\omega }\left(t\right),\chi f_{\omega }\left(\phi_{\omega }\right)\right]+d\left[\phi_{\omega }\left(t\right)+\chi f_{\omega }\left(\phi_{\omega }\right),\phi_{0}\right].$$

Next, we have that

$$\left(g\left(t+h\right)-g\left(t\right)\right)\chi^{-1}\leq (d\left[\phi_{\omega }\left(t+\chi \right)-\phi_{\omega }\left(t\right),\chi f_{\omega }\left(\phi_{\omega }\right)\right]$$

$$+d\left[\phi_{\omega }\left(t\right)+\chi f_{\omega }\left(\phi_{\omega }\right),\phi_{0}+\chi f_{\omega }\left(\phi_{0}\right)\right]-d\left[\phi_{\omega },\phi_{0}\right]+d\left[\chi f_{\omega }\left(\phi_{0}\right),\theta_{0}\right])\chi^{-1}.$$

From condition (1) of Theorem 5 and above relation, we have

$$D^{+}g\left(t\right)\leq \lim_{\chi \to 0^{+}}d\left[\frac{\phi_{\omega }\left(t+\chi \right)-\phi_{\omega }\left(t\right)}{\chi },f_{\omega }\left(\phi_{\omega }\left(t\right)\right)\right]$$

$$+\lim\sup\left(d\left[\phi_{\omega }\left(t\right)+\chi f_{\omega }\left(\phi_{\omega }\left(t\right)\right),\phi_{0}+\chi f_{\omega }\left(\phi_{0}\right)\right]\right)\chi^{-1}$$

$$-d\left[\phi_{\omega },\phi_{0}\right]+d\left[f_{\omega }\left(\phi_{0}\right),\theta_{0}\right]\leq -\beta \left[\phi_{\omega }\left(t\right),\phi_{0}\right]+d\left[f_{\omega }\left(\phi_{0}\right),\theta_{0}\right]$$

$$=-\beta g\left(t\right)+d[f_{\omega }\left(\phi_{0}\right),\theta_{0}].$$

Condition (2) of Theorem 5 implies

$$d\left[f_{\omega }\left(\phi_{0}\right),\theta_{0}\right]\leq K_{\omega }\mathrm{for}t\in \left[0,c\right)\mathrm{and}\omega =\omega^{*}.$$

For $\omega =\omega^{*}$ and some values s,t∈[0,c), s<t, we have

$$d\left[\phi_{\omega }\left(t\right),\phi_{\omega }\left(s\right)\right]\leq d\left[\phi_{0}+\int_{t_{0}}^{t}\phi_{\omega }\left(\tau \right)d\tau ,\phi_{0}+\int_{0}^{s}\phi_{\omega }\left(\tau \right)d\tau \right]$$

$$\leq \int_{s}^{t}d\left[f_{\omega }\left(\phi_{\omega }\left(\tau \right)\right),\theta_{0}\right]d\tau \leq K_{\varkappa }\left(t-s\right).$$

Therefore, the limit $\lim_{t\to c^{-}}\phi_{\omega }\left(t\right)$ exists, which contradicts our assumption on the continuability of $\phi_{\omega }\left(t\right)$ to the right of *c*. Hence, the solution $\phi_{\omega }\left(t\right)$ is defined on [0,∞) for $\omega =\omega^{*}$.

Finally, we will prove the uniqueness of the solution $\phi_{\omega }\left(t\right)$ for $\omega =\omega^{*}$. Consider the solutions $\phi_{\omega }\left(t\right)$ and $\gamma_{\omega }\left(t\right)$ for $\phi_{\omega }\left(0\right)=\gamma_{\omega }\left(0\right)=\phi_{0}$. We apply Condition (1) of Theorem 5 and obtain

(27) $$d\left[\phi_{\omega }\left(t\right),\gamma_{\omega }\left(t\right)\right]\leq d\left[\phi_{\omega }\left(0\right),\gamma_{\omega }\left(0\right)\right]\exp\left(-\beta t\right)$$

for t≥0 and $\omega =\omega^{*}$, which implies the uniqueness. The proof is completed. □

For $\omega =\omega^{*}$, let’s assume that

$$f_{\omega }\left(\phi \right)+\phi =S_{\omega }\left(\phi \right),\;S_{\phi }\in C\left(E^{n},E^{n}\right).$$

Then, we will establish the following result.

**Theorem** **6.** Let for the family of fuzzy differential Equation (26) with right-hand sides $S_{\omega }\left(\phi \right)$ all conditions of Theorem 5 hold for $\omega =\omega^{*}$. Then, there exists $\phi_{\omega }^{*}\in E^{n}$ such that $S_{\omega }\left(\phi_{\omega }^{*}\right)=\phi_{\omega }^{*}$ for $\omega =\omega^{*}$.

**Proof.**  Since the differential Equation (26) are autonomous, the mappings $T_{\omega }\left(t\right)\phi_{0}=\phi_{\omega }\left(t,\phi_{0}\right)$ for t≥0 and $\omega =\omega^{*}$. Also, $T_{\omega }\left(t\right)$ is a one-parametric family with semigroup properties. From (27), we obtain

$$d\left[\phi_{\omega }\left(t,\phi_{0}\right),\gamma_{\omega }\left(t,\gamma_{0}\right)\right]\leq d\left[\phi_{0},\gamma_{0}\right]\exp\left(-\beta t\right),t\geq 0,\omega =\omega^{*},$$

or

(28) $$d\left[T_{\omega }\left(t\right)\phi_{0},T_{\omega }\left(t\right)\gamma_{0}\right]\leq d\left[\phi_{0},\gamma_{0}\right]\exp\left(-\beta t\right),t\geq 0,\omega =\omega^{*}.$$

Let $t^{*}>0$ be such that $\exp\left(-\beta t^{*}\right)<\frac{1}{2}$. Then, from (28), we obtain

(29) $$d\left[T_{\omega }\left(t\right)\phi_{0},T_{\omega }\left(t\right)\gamma_{0}\right]\leq \frac{1}{2}d\left[\phi_{0},\gamma_{0}\right].$$

The estimate (29) implies that $T_{\omega }\left(t\right)$ is contractive. Therefore, there exists a $\phi_{\omega }^{*}$ such that $T_{\omega }\left(t^{*}\right)\phi_{\omega }^{*}=\phi_{\omega }^{*}$ for $\omega =\omega^{*}$. It is trivial to show that $\phi_{\omega }^{*}$ is a fixed point for the mapping $T_{\omega }\left(t\right)$ for all t≥0. Since $T_{\omega }\left(t\right)$ and $T_{\omega }\left(t^{*}\right)$ are commutative, then

$$d\left[T_{\omega }\left(t\right)\phi_{\omega }^{*},\phi_{\omega }^{*}\right]=d\left[T_{\omega }\left(t^{*}\right)T_{\omega }\left(t\right)\phi_{\omega }^{*},T_{\omega }\left(t^{*}\right)\phi_{\omega }^{*}\right]\leq \frac{1}{2}d\left[T_{\omega }\left(t\right)\phi_{\omega }^{*},\phi_{\omega }^{*}\right].$$

From the above relation, we have that $T_{\omega }\left(t\right)\phi_{\omega }^{*}=\phi_{\omega }^{*}$ for $t\geq t_{0}$, and hence, $f_{\omega }\left(\phi_{\omega }^{*}\right)=\theta_{0}$ for $\omega =\omega^{*}$. Since $f_{\omega }\left(\phi \right)+\phi =S_{\omega }\left(\phi \right)$, we conclude that $S_{\omega }\left(\phi_{\omega }^{*}\right)=\phi_{\omega }^{*}$ for $\omega =\omega^{*}$, which proves Theorem 6. □

## 9. Stability Concept

In this section, the stability of the equilibrium state $\theta_{0}\in E^{n}$ of the family of regularized differential Equation (2) will be analyzed based on a generalized comparison principle. To this end, we introduce the following definition.

**Definition** **3.** *The steady state $\theta_{0}\in E^{n}$ of the family of differential Equation (2) is said to be stable. If for any ε>0 and $t_{0}\in R_{+}$, there exists $\delta (t_{0},\varepsilon )>0$ such that $d\left[\phi_{0},\theta_{0}\right]<\delta \left(t_{0},\varepsilon \right)$ implies $d[\phi \left(t\right),\theta_{0}]<\varepsilon$ for any $t\geq t_{0}$ and any value of ω∈[0,1].*

In order to apply the comparison strategy, we consider a family of scalar equations

(30) $$d\eta /dt=G_{\omega }\left(t,\eta \right),\;\eta \left(t_{0}\right)=\eta_{0}\geq 0,$$

where $G_{\omega }\in C\left(R_{+}\times R_{+},R\right)$ and $G_{\omega }\left(t,0\right)=0$ for $t\in R_{+}$ and ω∈[0,1].

**Definition** **4.** The trivial solution η=0 of (30) is said to be stable. If for any $\varepsilon^{*}\in \left(0,H\right)$ and $t_{0}\in R_{+}$, there exists $\delta^{*}=\delta^{*}\left(t_{0},\varepsilon^{*}\right)>0$ such that

$$\sigma_{\omega }\left(t,t_{0},\eta_{0}\right)<\varepsilon^{*}\;\mathrm{for}\;t\geq t_{0}$$

whenever $\eta_{0}<\delta^{*}$, where $\sigma_{\omega }\left(t,t_{0},\eta_{0}\right)$ is the family of maximal solutions of the IVP (30) defined on $t\geq t_{0}$ for any ω∈[0,1].

We will establish an inequality, which will be used below.

**Lemma** **3.** *Assume that:*

*(1) The functions $G_{\omega }\left(t,\eta \right)$ are continuous for $t_{0}\leq t\leq t_{0}+a$, ω∈[0,1] and |η|<∞.*

*(2) For any ω∈[0,1], the functions $G_{\varkappa }\left(t,\eta \right)$ is quasi-monotonic [35] and nondecreasing on η.*

*(3) For any ω∈[0,1], the family of maximal solutions $\sigma_{\omega }\left(t,t_{0},\eta_{0}\right)$ of the set of differential Equation (30) is defined for $t_{0}\leq t\leq t_{0}+a$.*

*(4) There exists a continuous function g(t), such that*

(31) $$g\left(t\right)\leq g\left(t_{0}\right)+\int_{t_{0}}^{t}G_{\omega }\left(s,g\left(s\right)\right)ds$$

*for $t\in [t_{0},t_{0}+a]$ and ω∈[0,1].*

*Then*

(32) $$g\left(t\right)\leq \sigma_{\omega }\left(t,t_{0},\eta_{0}\right)$$

*for $t\in [t_{0},t_{0}+a]$.*

**Proof.**  Denote by ϕ(t)

$$\phi \left(t\right)=g\left(t_{0}\right)+\int_{t_{0}}^{t}G_{\omega }\left(s,g\left(s\right)\right)ds.$$

for any value of ω∈[0,1].

Hence

g(t)≤ϕ(t)

and

$$d\phi /dt=G_{\omega }\left(t,g\left(t\right)\right).$$

For ω∈[0,1] condition (2) of Lemma 3 implies

$$d\phi /dt\leq G_{\omega }\left(t,\phi \left(t\right)\right)$$

for $t\in [t_{0},t_{0}+a]$. The comparison principle (cf. Theorem 1.5.4 in Ref. [35]) leads to the estimate

$$g\left(t\right)\leq \sigma_{\omega }\left(t,t_{0},\eta_{0}\right)$$

for $t\in [t_{0},t_{0}+a]$ and ω∈[0,1]. □

The following Corollary follows directly from Lemma 3.

**Corollary** **2.** *If in Lemma 3 the inequality (31) is in the form*

$$g\left(t\right)\leq f\left(t\right)+\int_{t_{0}}^{t}G_{\omega }\left(s,g\left(s\right)\right)ds,t\in \left[t_{0},t_{0}+a\right],\omega \in \left[0,1\right],$$

*where f(t) is a continuous function for $t\in [t_{0},t_{0}+a]$, then the estimate (32) takes the form*

$$g\left(t\right)\leq f\left(t\right)+\sigma^{*}\left(t,t_{0},0\right),$$

where $\sigma^{*}\left(t,t_{0},0\right)=\max_{\varkappa }\sigma_{\omega }^{*}\left(t,t_{0},0\right)$ and $\sigma_{\omega }^{*}\left(t,t_{0},0\right)$ is the family of solutions of the differential equations

$$d\zeta /dt=G_{\omega }\left(t,f\left(t\right)+\zeta \right),\zeta \left(t_{0}\right)=0,$$

*defined on $\left[t_{0},t_{0}+a\right]$ for any ω∈[0,1].*

The following result will present stability criteria for the steady state $\theta_{0}$ of the family of regularized differential Equation (2).

**Theorem** **7.** *Assume that:*

*(1) For any ω∈[0,1], the mapping $f_{\omega }\in C\left(R_{+}\times B\left(\rho \right),E^{n}\right)$, $B\left(\rho \right)=\{\phi \in E^{n}:d\left[\phi ,\theta_{0}\right]<\rho \}$, $f_{\omega }\left(t,\theta_{0}\right)=\theta_{0}$ for all $t\geq t_{0}$.*

*(2) There exists a family of functions $G_{\omega }\left(t,\eta \right)$ that satisfies the conditions of Lemma 3 and are such that*

$$\lim_{\chi \to 0^{+}}\sup\left\{\left(d\left[\phi +\chi F_{\omega }\left(t,\eta \right),\theta_{0}\right]-d\left[\phi ,\theta_{0}\right]\right)\chi^{-1}\right\}\leq G_{\omega }\left(t,d\left[\phi ,\theta_{0}\right]\right)$$

*for all $t\in R_{+}$, ϕ∈B(ρ) and ω∈[0,1].*

*Then, the stability properties of the zero solution of the family of comparison Equation (30) imply the corresponding stability properties of the stationary state $\theta_{0}$ of the family of fuzzy differential Equation (2).*

**Proof.**  Let $\varepsilon^{*}\in \left(0,H\right)$ and $t_{0}\in R_{+}$ be given, and let the trivial solution y=0 of the set of comparison Equation (30) be stable. Denote $g\left(t\right)=d[\phi \left(t\right),\theta_{0}]$, and from condition (2) of Theorem 7, we have

(33) $$D^{+}g\left(t\right)\leq G_{\omega }\left(t,g\left(t\right)\right)$$

for all $t\in R_{+}$ and ω∈[0,1].

The inequality (33) and Lemma 3 imply

$$g\left(t\right)\leq \sigma_{\omega }\left(t,t_{0},\eta_{0}\right)$$

for $t\geq t_{0}$ and ω∈[0,1].

From the stability of the state η=0 of the family of Equation (30), for the given $\varepsilon^{*}>0$ and $t_{0}$, there exists $\delta^{*}=\delta^{*}\left(t_{0},\varepsilon^{*}\right)>0$ such that $\eta_{0}<\delta^{*}$ implies $\sigma_{\omega }\left(t,t_{0},\eta_{0}\right)<\varepsilon^{*}$ for all $t\geq t_{0}$ and ω∈[0,1].

We will show that if $d\left[\eta_{0},\theta_{0}\right]<\delta^{*}$, then $d[\eta \left(t\right),\theta_{0}]<\varepsilon$ for all $t\geq t_{0}$ and ω∈[0,1], where $\varepsilon <\varepsilon^{*}$. If this is not true, there exists a solution $\tilde{\eta }\left(t\right)=\tilde{\eta }\left(t,t_{0},\eta_{0}\right)$ of the IVP (2) with $d\left[\eta_{0},\theta_{0}\right]<\delta^{*}$, and a $t_{1}>t_{0}$ such that

$$d\left[\tilde{\eta }\left(t_{1}\right),\theta_{0}\right]=\varepsilon^{*}\mathrm{and}d\left[\tilde{\eta }\left(t\right),\theta_{0}\right]\leq \varepsilon <\varepsilon^{*}$$

for $t_{0}\leq t<t_{1}$. For $t=t_{1}$ we have

$$\varepsilon^{*}=d\left[\tilde{\eta }\left(t_{1}\right),\theta_{0}\right]<\sigma_{\omega }\left(t_{1},t_{0},\eta_{0}\right)<\varepsilon^{*},$$

which contradicts the assumption of existence of the point $t_{1}>t_{0}$. The contradiction proofs Theorem 7. □

**Remark** **4.** *Following the proof of Theorem 7, different stability properties of the regularized family of fuzzy differential Equation (2) can be proved.*

**Remark** **5.** *Section 9 offers stability results for the stationary state of the regularized family of fuzzy differential equations. These results can be also applied to other states of interest, such as periodic or almost periodic solutions. In such cases, appropriate fundamental results are necessary to guarantee their existence and uniqueness properties.*

**Remark** **6.** *In this paper, a new approach introduced in [2] is applied to investigate the properties of fuzzy systems of differential equations that include uncertain values of a parameter by means of the study of the corresponding properties of a regularized differential equation. The used technique and the results obtained have a wider applicability and can be further generalized. Also, the used methodology significantly simplifies the fundamental and qualitative analysis of such systems, which is illustrated by the next example.*

**Example** **1.** *In [2], we investigated some properties of the equilibrium (steady, stationary) state $\theta_{0}\in E^{n}$ of the following family of differential equations*

(34) $$\frac{d\psi }{dt}=F_{\omega }\left(t,\psi \right)+G\left(t,\psi ,\lambda \right),$$

*where $G\left(t,\psi ,\lambda \right)=F\left(t,\psi ,\lambda \right)-F_{\omega }\left(t,\psi \right)$ for all λ∈P. We also suppose that $F_{\omega }\in C\left(R_{+}\times E^{n},E^{n}\right)$ for all ω∈[0,1] and $G\in C(R_{+}\times E^{n}\times P,E^{n})$, $F_{\omega }\left(t,\theta_{0}\right)=G\left(t,\theta_{0},\lambda \right)=\theta_{0}$ for all $t\geq t_{0}$.*

*Let $F_{\omega }\left(t,\psi \right)$ and G(t,ψ,λ) be such that there exists p>1 and continuous positive functions K(t) and k(t) for all t∈I, for which:*

(1) *$d\left[F_{\omega }\left(t,\psi \right),\theta_{0}\right]\leq K\left(t\right)d\left[\psi ,\theta_{0}\right]$ for all ω∈[0,1];*
(2) *$d\left[G\left(t,\psi ,\lambda \right),\theta_{0}\right]\leq k\left(t\right)d^{p}\left[\psi ,\theta_{0}\right]$ for all λ∈P;*

(3) *$d\left[\psi_{0},\theta_{0}\right]<\delta \left(t_{0},\varepsilon \right)$;*
(4) *$v\left(t_{0},t\right){|}_{\delta }=\left(p-1\right)\delta^{p-1}\int_{t_{0}}^{t}k\left(s\right)\exp\left[\left(p-1\right)\int_{t_{0}}^{s}K\left(\tau \right)d\tau \right]ds<1$ for $t\geq t_{0}$;*
(5) *$\exp\left(\int_{t_{0}}^{t}K\left(s\right)ds\right){\left(1-v\left(t_{0},t\right){|}_{\delta }\right)}^{-\frac{1}{p-1}}<\frac{\varepsilon }{\delta }$ for $t\geq t_{0}$*

*whenever*

(35) $$d[\psi \left(t\right),\theta_{0}]<\varepsilon$$

*for $t\geq t_{0}$.*

*Under Conditions (1)–(5), the deviation of the solution ψ(t) from the state $\theta_{0}\in E^{n}$ is determined by*

(36) $$d\left[\psi \left(t\right),\theta_{0}\right]\leq \delta \left(t_{0},\varepsilon \right)\exp\left(\int_{t_{0}}^{t}K\left(s\right)ds\right){\left(1-v\left(t_{0},t\right){|}_{\delta }\right)}^{-\frac{1}{p-1}}$$

*for $t\geq t_{0}$.*

*Conditions (1), (2), and (4) imply that the estimate (36) holds for $t\in [t_{0},a]$.*

*From Conditions (3), (5), and (36) we obtain (35), which implies the stability of the state $\theta_{0}\in E^{n}$ of the family of fuzzy differential Equation (34).*

**Remark** **7.** *If Conditions (4) and (5) are satisfied uniformly with respect to $t_{0}\in R_{+}$ then (36) holds uniformly on $t_{0}$, and in this case, δ can be chosen independent on $t_{0}$, which implies that the state $\theta_{0}\in E^{n}$ of (34) will be uniformly stable.*

**Remark** **8.** *The proposed example shows the feasibility of the proposed approach and the results obtained. Since the obtained criteria are in the form of inequalities, they can be easily applied.*

## 10. Concluding Remarks

In this paper, new sufficient conditions for the existence of a number of fundamental properties of solutions of fuzzy differential equations are established on the basis of a new approach proposed in [2]. This approach is based on the regularization of an initial fuzzy differential equation with respect to the uncertain parameter. The stability of the steady state of the regularized system is also considered using the comparison method and nonlinear integral inequalities. The key outcomes of the proposed research can be summarized as:

- Sufficient conditions are established for the correctness of the regularization process;
- For the regularized differential equation criteria for the convergence of successive approximations, continuity and global existence of solutions are provided;
- The existence of an approximate solution is also investigated;
- Criteria for the existence of weak solutions of families of autonomous fuzzy differential equations are obtained;
- The stability notion for the family of regularized fuzzy differential equations is developed and analyzed.

The results obtained in this article are the basis for a qualitative analysis of fuzzy systems of differential equations containing delays and impulsive (impact) disturbances, modeling processes, and phenomena of the real world (see [36,37,38,39] and the bibliography there).

## Data Availability

Not applicable.
